# Functional expression of CLIFAHDD and IHPRF pathogenic variants of the NALCN channel in neuronal cells reveals both gain- and loss-of-function properties

**DOI:** 10.1038/s41598-019-48071-x

**Published:** 2019-08-13

**Authors:** Malik Bouasse, Hathaichanok Impheng, Zoe Servant, Philippe Lory, Arnaud Monteil

**Affiliations:** 0000 0001 2097 0141 grid.121334.6 IGF, CNRS, INSERM, University of Montpellier, LabEx ‘Ion Channel Science and Therapeutics’, Montpellier, France

**Keywords:** Channelopathies, Ion channels in the nervous system, Excitability, Membrane potential

## Abstract

The excitability of neurons is tightly dependent on their ion channel repertoire. Among these channels, the leak sodium channel NALCN plays a crucial role in the maintenance of the resting membrane potential. Importantly, NALCN mutations lead to complex neurodevelopmental syndromes, including infantile hypotonia with psychomotor retardation and characteristic facies (IHPRF) and congenital contractures of limbs and face, hypotonia and developmental delay (CLIFAHDD), which are recessively and dominantly inherited, respectively. Unfortunately, the biophysical properties of NALCN are still largely unknown to date, as well as the functional consequences of both IHPRF and CLIFAHDD mutations on NALCN current. Here we have set-up the heterologous expression of NALCN in the neuronal cell line NG108-15 to investigate the electrophysiological properties of NALCN carrying representative IHPRF and CLIFAHDD mutations. Several original properties of the wild-type (wt) NALCN current were retrieved: mainly carried by external Na^+^, blocked by Gd^3+^, insensitive to TTX and potentiated by low external Ca^2+^ concentration. However, we found that this current displays a time-dependent inactivation in the −80/−40 mV range of membrane potential, and a non linear current-voltage relationship indicative of voltage sensitivity. Importantly, no detectable current was recorded with the IHPRF missense mutation p.Trp1287Leu (W1287L), while the CLIFAHDD mutants, p.Leu509Ser (L509S) and p.Tyr578Ser (Y578S), showed higher current densities and slower inactivation, compared to wt NALCN current. This study reveals that heterologous expression of NALCN channel can be achieved in the neuronal cell line NG108-15 to study the electrophysiological properties of wt and mutants. From our results, we conclude that IHPRF and CLIFAHDD missense mutations are loss- and gain-of-function variants, respectively.

## Introduction

The maintenance of the neuronal resting membrane potential (RMP) and its modulation by hormones and neurotransmitters is a finely tuned process that involves a complex interplay between several classes of proteins including ion channels, exchangers, transporters, and G protein-coupled receptors^1,2^. Of importance, Lu and colleagues, in 2007, found that the sodium (Na^+^)-leak channel NALCN that conducts a background sodium influx critically regulates the RMP of neurons^3^. Disruption of NALCN expression in mice led to a ≈15 mV hyperpolarization of the RMP of hippocampal neurons and a decrease of their firing rate^3^. Of note, the homozygous knockout mice died within 24 h after birth as a consequence of a disrupted respiratory rhythm. The importance of NALCN in setting-up the neuronal RMP was then confirmed in other types of neurons and other species, showing a conserved role across species^4–11^. In addition, NALCN was also found to be involved in the modulation of the neuronal RMP by hormones and neurotransmitters. Indeed, it was demonstrated that NALCN channel could be activated by substance P, neurotensin, and acetylcholine^8,11–14^. This positive modulation of NALCN involves the corresponding G-protein coupled receptors through a G-protein-independent, src family of tyrosine kinase-dependent pathway^12,13^. Conversely, a negative modulation of NALCN activity was reported by activation of the the G-protein-coupled calcium-sensing receptor^15^, the GABA-B receptor^16^ as well as the Dopamine D2 receptor^16^. This NALCN inhibition involves a G-protein-dependent pathway^15,16^.

Both recessive and dominant pathogenic variants of human NALCN and its Unc80 ancillary subunit were recently described in several clinical and genetic studies^17–43^. Recessive pathogenic variants of NALCN and Unc80 are linked to a syndrome refered to as infantile hypotonia with psychomotor retardation and characteristic facies (IHPRF) type 1 (OMIM #615419) and type 2 (OMIM #616801), respectively. Dominant pathogenic variants of NALCN are responsible for a syndrome refered to as congenital contractures of limbs and face, hypotonia and developmental delay (CLIFAHDD; OMIM #616266). Both IHPRF and CLIFAHDD patients exhibit complex clinical traits of variable severity that may cause premature death in some cases. Interestingly, IHPRF and CLIFAHDD patients exhibit common symptoms such as hypotonia, facial dismorphisms, global developmental delay, constipation, and respiratory defects. Most, but not all, of the recessive IHPRF variants are predicted to result in truncated and non-functional proteins resulting in a loss-of-function phenotype. However, the electrophysiological and functional consequences of the IHPRF mutations are not known to date. Similarly, there is no functional data regarding the CLIFAHDD mutations. Of interest, all the CLIFAHDD mutations are missense and localize in the pore-forming region of NALCN, except the R1181Q mutation that localizes in the intracellular loop linking the transmembrane domains III and IV of NALCN.

Because the functional properties of recombinant NALCN are poorly known, we set-up heterologous expression of human NALCN in a neuron-like environment in order to investigate the electrophysiological properties of the wt, and the IHPRF and CLIFAHDD pathogenic variants of NALCN. We describe here that NALCN currents could be recorded in the neuronal cell line NG108-15 transfected with human NALCN constructs. The overexpressed NALCN current exhibits a time-dependent current decay that resembles inactivation. We also report that the W1287L IHPRF variant resulted in a non-functional NALCN channel, while the L509S and Y578S CLIFAHDD variants promote NALCN currents larger than the wt NALCN current. Importantly, both L509S and Y578S mutations impaired the inactivation kinetics of the NALCN currents, compared to wt NALCN channel. This study provides the first description of the functional consequence of the pathogenic NALCN missense mutations and suggests that loss and gain of NALCN activity supports the inheritance patterns of IHPRF and CLIFAHDD, respectively.

## Materials and Methods

### DNA constructs

The pcDNA3-GFP, pcDNA3-eGFP-hNALCN-wt, pcDNA3-eGFP-hNALCN-L509S, and the pcDNA3-eGFP-hNALCN-Y578S plasmids were previously described^13,21^. The pcDNA3-eGFP-hNALCN-W1287L plasmid was generated from the pcDNA3-eGFP-hNALCN-wt plasmid by using a site-directed mutagenesis standard protocol. The W1287L mutation was originally reported in^17^. The pRK5-mNLF1, pRK5-mNLF1-mCherry, and the pRK5-mNLF1-eGFP plasmids were kind gifts from Drs. M. Zhen and K. Aoyagi.

### Cell culture

NG108-15 cells (ATCC^®^ HB-12317^™^) were grown in Dulbecco’s modified Eagle’s medium (DMEM) supplemented with GlutaMax, 10% dialyzed fetal calf serum, 2% hypoxanthine/aminopterin/thymidine, and penicillin/streptomycin 1% (Invitrogen, Thermo Fisher Scientific). Cells with 70–80% of confluency were transfected in 35 mm culture dishes by using the JetPEI reagent (QBiogen, #101-40N) according to the manufacturer’s procedure. 24 h after transfection, cells were dissociated with Versene (Thermo Fisher Scientific, #15040-033) and plated at a dilution of 1:100 per 35 mm Petri dish for electrophysiological recordings. Differentiation of NG108-15 cells into neuron-like cells was then induced during 5 days by replacing the culture media by the same medium with lowered serum concentration to 1% and the addition of dbcAMP 200 µM (Sigma-Aldrich, #D0627) and dexamethasone 0.1 µM (Sigma-Aldrich, #D4902). Analysis of NALCN expression (PCR, Western-blot and patch-clamp experiments) was performed 6 to 7 days post-transfection. The control condition (Mock) corresponds to cells transfected with NLF1 alone.

### Non-quantitative (nqPCR) and quantitative PCR (qPCR)

Total RNA was extracted from cell cultures and rat tissues using the RNeasy Plus universal mini kit (Qiagen #74134). One μg of total RNA were reverse transcribed using the iScript kit (BioRad #1708891) according to the manufacturer’s protocol. For nqPCR, Taq polymerase (New England Biolabs #M0273S) and dNTP (New England Biolabs #N0447S) were used according to the manufacturer’s protocol with the following parameters: 94 °C-3min, 30 cycles of 94 °C-45sec, 55 °C-45sec, 68 °C-90sec, and a final step at 68 °C-10min. Primers used for nqPCR were: NALCN-sense 5′-TTCTTTGTTGGATGCCTCAAA-3′, NALCN-antisense 5′-GGCCCCACACGATGAATAAT-3′, Unc79-sense 5′-TTGGATTTCCAGAGCAATCAA-3′, Unc79-antisense 5′-ACCTGGGCCATTTTGAACTG-3′, Unc80-sense 5′-AAGCTGGCACCATATGACAC-3′, Unc80-antisense 5′-ACAACAGCTGTGCTGAAGGG-3′, NLF1-sense 5′-GGCTCTTTTTCTAGGAAACTC-3′, NLF1-antisense 5′-CACGCTTTCAAACTCTTCGT-3′, APP-sense 5′-CAGGTCATGAGAGAATGGGA-3′, APP-antisense 5′-ACGGAGGTGTGTCATAACCT-3′. For qPCR, RT products were diluted 10 times with H_2_O and stored at −20 °C until use. Real-time PCR was performed in 384 well plates in a final volume of 10 μl using SYBR Green dye detection on the LightCycler480 system (Roche-Diagnostic #04887352001). The primer pairs were designed using Primer 3 input software, and their specificity and efficacy was experimentally validated. Cq for individual genes were determined using the second Derivative Max tool of the LightCycler480 software. The relative RNA expression was calculated using the RNA helicase Ddx17 as a reference gene. Relative expressions were calculated using the ΔΔCq method^44^. Primers used in this study were: NALCN-sense 5′-ccagagctagaagaaggctatca-3′, NALCN-antisense 5′-tgcctgctaagtcccaggt-3′, Unc80-sense 5′-ctctgtggatcgcctgtctt-3′, Unc80-antisense 5′-gacagcgctggtgaacttg-3′, Unc79-sense 5′-gccgaggaatcagaatttaaga-3′, Unc79-antisense 5′-gctgacggcaatcttcctt-3′, NLF1-sense 5′-aaccgaggcaagaacaacc-3′, NLF1-antisense 5′-gtctctaggcgccacacc-3′, Ddx17-sense 5′-aagttgatgcagcttgtgga-3′, Ddx17-antisense 5′-gaagtagtccggtatcgtgagc-3′.

### Biotinylation of proteins at the cell surface

Cells were washed twice with cold Phosphate Buffered Saline (PBS, Life Technologies #14040-091). For biotinylation, the cells were incubated in cold biotinylation solution (0.5 mg/ml EZ-Link Sulfo-NHS-SS-Biotin, Thermo Fisher Scientific #21331 in PBS) for 30 min on ice. The biotinylation solution was removed, and the cells were washed three times with stop solution (10 mM Tris-HCl, pH 7.4, 120 mM NaCl). Cells were lysed in lysis buffer (10 mM Tris-HCl, pH 7.4, 120 mM NaCl, 1% Triton-X-100 (v/v)) supplemented with proteases inhibitors (Roche Applied Science #04693124001) and centrifuged at 10,000 × *g* for 30 min at 4 °C. The clear supernatant was incubated with NeutrAvidin agarose (Thermo Fisher Scientific #29200) for 2 hours at 4 °C. After incubation, the beads were washed three times with lysis buffer and proteins were eluted by adding laemmli buffer then submitted to immunoblotting.

### Immunoblotting

Cells were lysed on ice for 20 min with NP-40 buffer containing 10 mM Tris-HCl, pH 7.4, 120 mM NaCl, 1% NP-40, and proteases inhibitors (Roche Applied Science #04693124001). Cell lysates were spun at 10,000 × *g* for 30 min at 4 °C. Sixty micrograms of proteins were mixed with a 4x Laemmli buffer and then loaded on SDS-PAGE. Proteins resolved onto 4–20% polyacrylamide gels (Bio-rad #4561096) were transferred to Hybond C nitrocellulose membranes (GE Healthcare #10600016). Membranes were immunoblotted with primary antibodies (rabbit anti-GFP, 1:10000, Torrey Pines Biolabs #TP401; rabbit anti-α-actinin, clone D6F6, 1:3000, Cell Signaling #6487; mouse anti-Na^+^/K^+^-ATPase, 1:5000, Abcam #AB7671; rabbit anti-calreticulin, 1:1000, Abcam #92516) and then with either an anti-rabbit or anti-mouse horseradish peroxidase (HRP)–conjugated secondary antibody (anti-rabbit, 1:10000, GE Healthcare #NA934; anti-mouse, 1:5000, Merck #12-349). Immuno-reactivity was detected with an enhanced chemiluminescence substrate for detection of HRP (SuperSignal West Pico Chemiluminescent Substrate, Thermo Fisher Scientific #34080).

### Electrophysiological recordings

Macroscopic currents were recorded at room temperature using an Axopatch 200B amplifier (Molecular Devices). Borosilicate glass pipettes had a resistance of 3 to 5 MΩ when filled with an internal solution containing (in mM): 150 CsCl, 5 HEPES, 2 MgCl_2_, 1.1 EGTA (pH adjusted to 7.34 with NaOH, ~300 mOsm). The extracellular solution contained (in mM): 142.6 NaCl, 2 CaCl_2_, 5.6 KCl, 0.8 MgCl_2_, and 10 HEPES, 5 Glucose (pH adjusted to 7.35 with NaOH, ~310 mOsm). In some experiments, when indicated, the CaCl_2_ concentration was reduced to 0.1 mM. The recording chamber was constantly perfused (~100 µl/min) with the control (Na^+^-containing) solution using a gravity-driven homemade perfusion device, which allowed extracellular medium change and drug application.

### Data analysis

Data were analyzed using pCLAMP9 (Molecular Devices) and GraphPad Prism (GraphPad) softwares. Results are presented as the mean ± SEM, and n is the number of cells. Statistical analysis was performed either with the Student *t*-test or a Mann-Withney test or with one-way ANOVA combined with a Tukey post-test for multiple comparisons (*p < 0.05, **p < 0.01, ***p < 0.001, ****p < 0.0001).

## Results

### The NG108-15 cell line endogenously expresses Unc79 and Unc80

**Figure 1:**
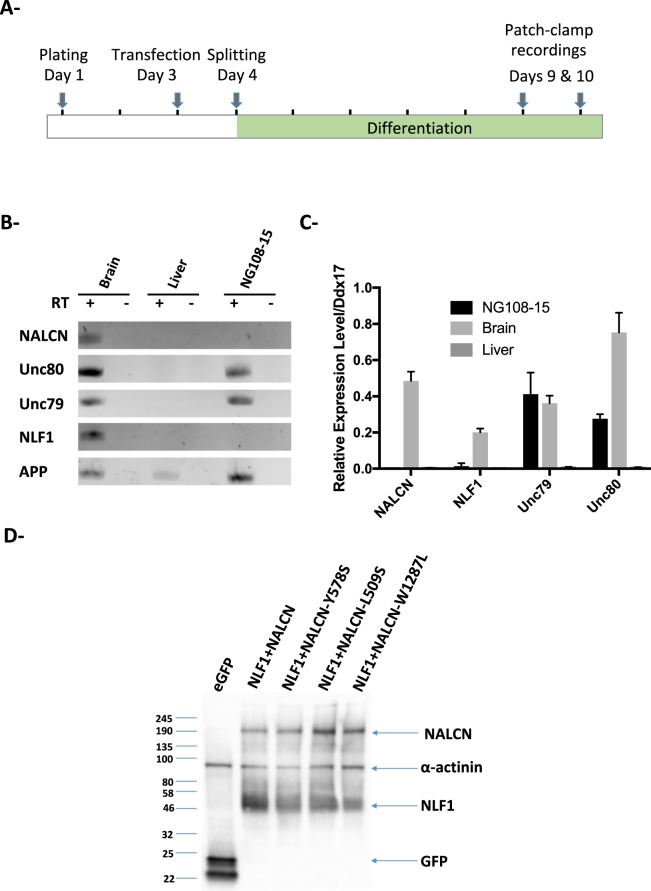
The NG108-15 cell line endogeneously expresses Unc79 and Unc80. (**A**) Schematic representation of the protocol used to achieve differentiation of NG108-15 cells into neuron-like cells. (**B**) RT-nqPCR with total RNA extracted from differentiated NG108-15 cells, rat adult brain and rat adult liver. The + and − symbols indicates the presence or the absence of the Reverse Transcriptase respectively. The ubiquitously expressed transmembrane Amyloid Precuror Protein (*App*)-encoding mRNA was used as an internal control. (**C**) RT-qPCR results with the same RNA samples than in B- showing that both *Unc79* and *Unc80*, but not *Nalcn* and *Nlf1*, are expressed in differentiated NG108-15 cells. The gene encoding the RNA helicase Ddx17 was used as a housekeeping gene (n = 4). (**D**) Representative western blot showing that transfection of the wt NALCN- and its pathogenic variants W1287L, Y578S, L509S, and NLF1-encoding cDNAs in NG108-15 cells results in a high expression level of the corresponding proteins, but not in the eGFP condition.

We previously demonstrated that the NG108-15 cell line is a suitable neuronal model for the study of voltage-gated calcium channels^45,46^. Importantly, NG108-15 cells can be differentiated into cholinergic neuron-like cells by adding dbcAMP and dexamethasone for 4–5 days (*see the* Materials and Methods section; Fig. 1A)^47^. In order to determine whether differentiated NG108-15 cells could be used to functionally express NALCN, we checked for the expression of the known components of the NALCN channelosome by RT-nqPCR and RT-qPCR (Fig. 1B,C). As a matter of fact, NALCN belongs to a large protein complex that includes, in addition to NALCN, at least 3 additional subunits: Unc79, Unc80 and NLF1 (*reviewed in*^48^). Both the Unc79- and the Unc80-encoding mRNAs were detected in differentiated NG108-15 cells, but not NALCN nor NLF1. However, because of the lack of commercially available antibodies, we were not able to confirm protein expression by Western blotting. We next examined by Western blot whether missing components of the NALCN channelosome (e.g. NALCN and NLF1) could be provided by transfection of ectopic cDNAs. We found that wt NALCN, and the pathogenic variants, as well as and NLF1 were expressed at high level in transfected NG108-15 cells 6 days after transfection and differentiation (Fig. 1D).

### Co-expression of NALCN and NLF1 in NG108-15 cells leads to the recording of a Na^+^ background current

**Figure 2:**
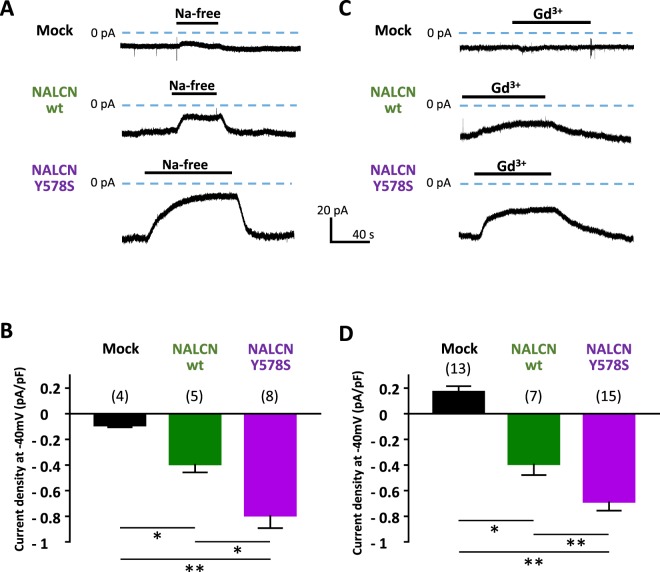
Transient expression of the wild-type (wt) NALCN and its CLIFAHDD variant Y578S together with NLF1 in differentiated NG108-15 cells results in the expression of a NMDG- and Gd^3+^-sensitive background Na^+^ current. (**A**) Representative traces obtained on cells held at −40 mV (HP) during application/wash of an extracellular Na^+^-free solution (replaced with NMDG) for a mock (NLF1)-transfected cell (top), a NALCN/NLF1 (wt)-transfected cell (middle), a NALCN CLIFAHDD mutant (Y578S)/NLF1-transfected cell (bottom). (**B**) Density of the external Na^+^-dependent background current (Na^+^-free) for mock-transfected cells (−0.088 ± 0.03 pA/pF, n = 4); NALCN: −0.391 ± 0.14 pA/pF, n = 5; NALCN-Y578S: −0.79 ± 0.32 pA/pF, n = 8). (**C**) Representative traces obtained on cells held at −40 mV during application/wash of Gd^3+^ (10 µM) for a mock-transfected cell (top), a NALCN (wt)-transfected cell (middle), a NALCN CLIFAHDD mutant (Y578S)-transfected cell (bottom). (**D**) Average density of the Gd^3+^-sensitive background current for mock-transfected cells (0.168 ± 0.16 pA/pF, n = 13), NALCN (wt)-transfected cells (−0,391 ± 0.23 pA/pF, n = 7) and NALCN-Y578S-transfected cells (−0.684 ± 0.27 pA/pF, n = 15). Both the Na^+^-dependent background current and the Gd^3+^-sensitive background current were significantly of higher density for wt NALCN and its pathogenic variant Y758S compared to the control condition (p = 0,03 and 0,004 for the Na^+^-dependent component and P < 0.0001 and <0.0001 for the Gd^3+^-sensitive component respectively). In addition, densities of the Na^+^-dependent background current and the Gd^3+^-sensitive background current were significantly lower for wt NALCN compared to its pathogenic variant Y578S (p = 0.045 and p = 0.029 respectively). p values were calculated with a Mann-Whitney statistical test.

Using the patch-clamp technique, whole-cell recordings were carried out in NG108-15 cells co-transfected with NALCN and NLF1. We first performed experiments to examine the effect of external Na^+^ removal (NMDG-substituted) when cells were held at −40mV. Of note, we did not observed any effect of Na^+^ removal on non-differentiated NG108-15 cells (*data not shown*). Replacing external Na^+^ by the impermeant cation NMDG in differentiated cells resulted in a significant decrease of a background current in cells co-transfected with NALCN and NLF1 (−0.391±0.14 pA/pF, n = 5), compared to the control condition (Mock; −0.088±0.03 pA/pF, n = 4, p = 0,031, Fig. 2A,B). Importantly, NMDG application revealed that this Na^+^ background current was even larger in cells expressing the CLIFAHDD variant NALCN-Y578S (−0.79±0.32 pA/pF, n = 8, p = 0.004) and was significant larger than wt NALCN (p = 0.045).

NALCN current was previously reported to be Gd^3+^-sensitive and TTX-resistant^3,12,13^. We therefore studied the effect of Gd^3+^ and TTX on the background current in NALCN-transfected NG108-15 cells. We found that the background current in cells expressing wt NALCN and its pathogenic variant Y578S was inhibited by Gd^3+^ (10 μM; Fig. 2C,D). In addition, we found no effect of TTX (10 μM) on the background Na^+^ current in NALCN-transfected NG108-15 cells (*data not shown*). Taken together, these data indicate that expression of NALCN channel results, in differentiated NG108-15 cells, in the functional expression of a Na^+^ background current with pharmacological properties reminiscent to those described previously for NALCN currents^3,12,13^.

### The Na^+^ background current amplitude is differentially affected by the CLIFAHDD and IHPRF1 variants of NALCN

**Figure 3:**
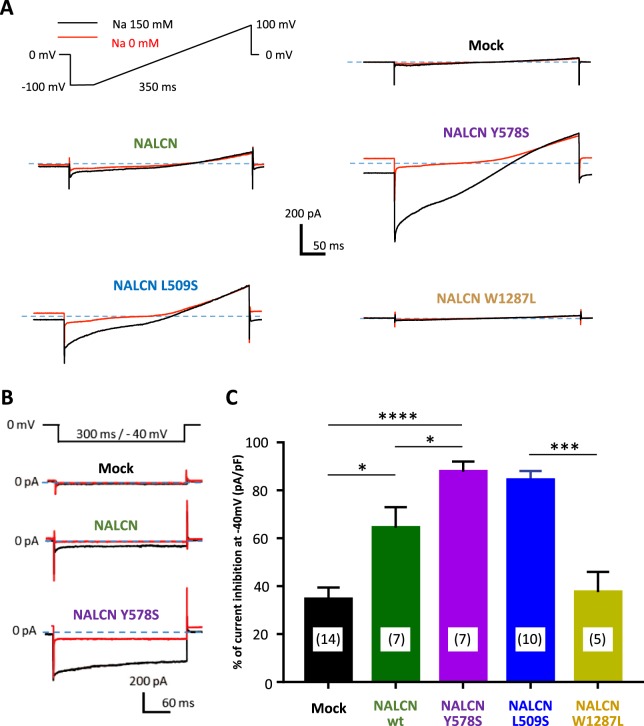
Amplitude of the Na^+^-dependent component of the NALCN current carried out by the CLIFAHDD variants (Y578S, L509S) and the IHPRF variant (W1287L) of the NALCN channel. (**A**) Representative current traces elicited by a voltage ramp protocol (from −100 mV to +100 mV) in the presence (black traces) and in the absence (red traces) of extracellular Na^+^. (**B**) Representative current traces elicited by a voltage step protocol (HP 0 mV, test pulse (TP) at −40 mV) in the presence (black traces) and in the absence (red traces) of extracellular Na^+^. (**C**) Percentage of current inhibition obtained in Na^+^-free condition for mock-transfected cells (34.51 ± 18.37%, n = 14), for NALCN-transfected cells (64.41 ± 22.67%, n = 7), for NALCN-Y578S-transfected cells (87.81 ± 11.2%, n = 7), NALCN-L509S-transfected cells (84.25 ± 12.06%, n = 10) and for NALCN-W1287L-transfected cells (37.56 ± 12.06%, n = 5). Compared to the control condition, the percentage of inhibition was significantly larger for NALCN-wt (p = 0.026), NALCN-Y578S (p < 0.0001), NALCN-L509S (p < 0.0001) but not for NALCN-W1287L (p = 0.864). A significant difference in the percentage of inhibition was also found for NALCN-wt compared to its pathogenic variant Y578S (p = 0.017) but not for L509S (p = 0.157) and W1287L (p = 0.197). P values were calculated with a Mann-Whitney statistical test.

The Na^+^-dependence of the background current in NALCN-transfected NG108-15 cells was further investigated using voltage ramp and voltage step protocols (Fig. 3). Removal of external Na^+^ revealed that the CLIFAHDD variants Y578S and L509S conducted a higher Na^+^ background current compared to wt NALCN. Using a voltage ramp command from −100 mV to +100 mV (Fig. 3A), we observed that the inward Na^+^ background current was rather linear in a wide range of membrane potentials (−100 to +100 mV) for both wt NALCN and its CLIFAHDD variants. Importantly, no detectable inward Na^+^ background current was recorded in cells mock-transfected (NLF1 alone) or co-transfected with the IHPRF1 W1287L NALCN mutant. Amplitude of the inward Na^+^ background current was also measured using one membrane voltage step from 0 mV to −40 mV (Fig. 3B) and normalized by calculating, for each cell, the percentage of inhibition of this current due to the removal of the external Na^+^ (Fig. 3C). The small current recorded in mock- and the IHPRF1 mutant-transfected cells was weakly reduced in the absence of external Na^+^ (34.52 ± 18.37%, n = 15, and 37.56 ± 18.70%, n = 5, respectively). Reduction of the Na^+^ background current was higher in wt NALCN- and the two CLIFAHDD missense mutants (Y578S; L509S)-transfected cells: 64.41 ± 22.67%, n = 7, p = 0.026 for wt, 87.81 ± 12.06%, n = 7, p < 0.0001, for Y578S and 84.25 ± 12.06% n = 10, p < 0.0001, for L509S, NALCN channels. These data clearly indicate that wt NALCN channel generates Na^+^ background current in the neuronal NG108-15 cells. This Na^+^ background current is significantly larger for the two CLIFAHDD mutants investigated here, especially the Y578S variant. On the contrary, no Na^+^ background current could be detected in cells expressing NALCN channels carrying the IHPRF1 missense variant W1287L (p = 0.864).

### Current-Voltage relationship of wt and mutant Y578S NALCN currents in differentiated NG108-15 cells

**Figure 4:**
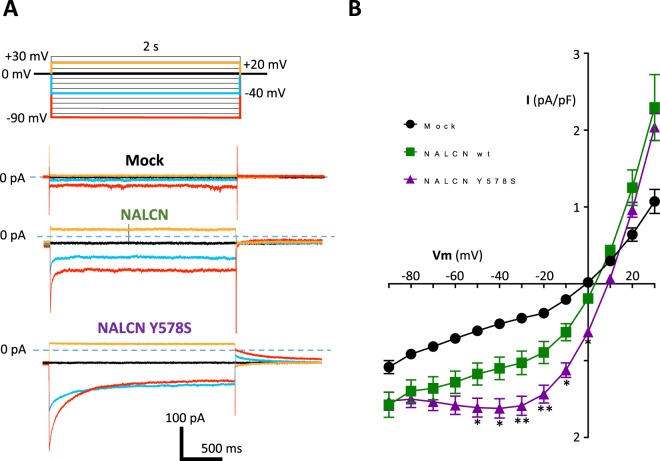
Current-voltage (I/V) relationship of the steady-state NALCN current for wild-type (wt) and its CLIFAHDD variant Y578S in differentiated NG108-15 cells. (**A**) Representative current traces elicited by a membrane voltage step protocol (test pulses from −90 mV to + 30 mV) from a HP at 0 mV, for a mock-transfected cell (top), a NALCN (wt)-transfected cell (middle), a NALCN (Y578S)-transfected cell (bottom). Only the current traces obtained for the test-pulses at −90 mV (in red), −40 mV (in blue), 0 mV (in black) and +20 mV (in orange) are shown. (**B**) Corresponding I/V relationships for mock transfected cells (black circles, n = 44), NALCN wt (green squares, n = 45) and its pathogenic variant NALCN Y578S (purple triangles, n = 73). Statistical significance was calculated with a Tukey’s multiple comparisons test.

A current-voltage (I/V) step protocol was used to characterize further the electrophysiological properties of the wt and Y578S CLIFAHDD mutant NALCN channels (Fig. 4). Using hyperpolarizing steps from a holding potential (HP) 0 mV, we found that both wt and Y578S NALCN currents displayed a time-dependent inactivation-like decay (Fig. 4A). In addition, our measurements of the steady-state NALCN current (at 2 s) revealed a non-linear I/V relationship for both wt and Y578S NALCN current in NG108-15 cells (Fig. 4B). Similar findings were made with the mutant NALCN L509S (*data not shown*). Altogether, here we describe that NALCN channels heterogously expressed in differentiated NG108-15 cells exhibit electrophysiological properties indicative of a voltage sensitivity. This finding is novel, considering previous studies that reported a linear, ohmic-like, I/V relationship for the NALCN current, as well as a voltage-independent time-course of the NALCN current^3,4,15,32,49–51^.

### Voltage-dependent inactivation of the NALCN current in differentiated NG108-15 cells

**Figure 5:**
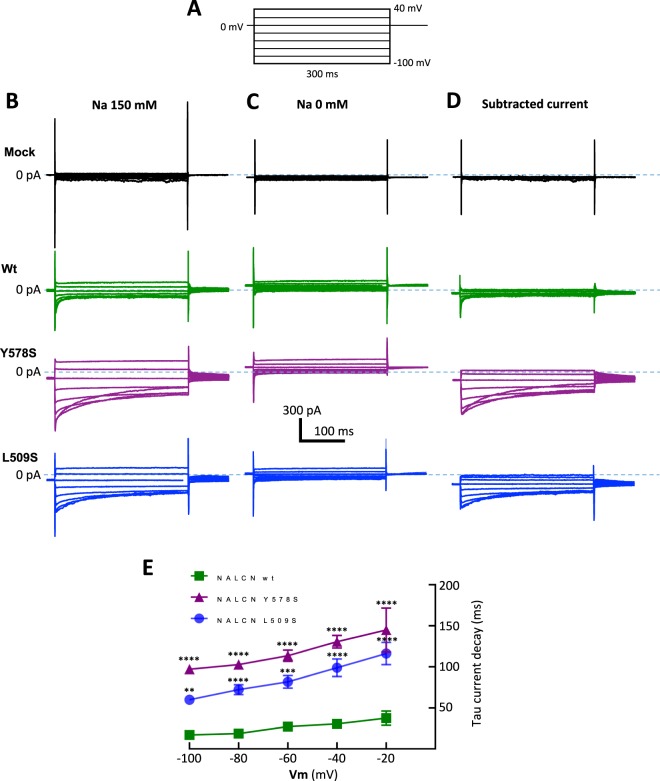
Time- and voltage-dependent inactivation of the NALCN current elicited by the wt, Y578S and L509S variants. (**A**) Voltage step protocol used to elicit representative current traces obtained in the presence of extracellular Na^+^ (**B**), in the absence of extracellular Na^+^ (**C**) and the corresponding trace subtraction to isolate the Na^+^-dependent component of the NALCN current (**D**) for mock-transfected cells (black traces), NALCN-wt-transfected cells (green traces), NALCN-Y578S-transfected cells (purple traces) and NALCN-L509S-transfected cells (blue traces). (**E**) Inactivation kinetics (time-constant, τ) of the Na^+^-dependent component of the wt (n = 7), NALCN-Y578S (n = 7) and NALCN-L509S (n = 6) currents in the −100 mV to −20 mV range. Statistical significance was calculated with a Tukey’s multiple comparisons test.

To determine whether the inactivation-like process was readily supported by NALCN channel activity, we examined its dependence to the external Na^+^ concentration (Fig. 5). The Na^+^-dependent component (subtracted current, Fig. 5D) of wt NALCN current (green traces), the L509S mutant (blue traces) and the Y578S mutant (purple traces) was isolated by substracting current traces obtained in the presence of extracellular Na^+^ (Fig. 5B) to the current traces obtained when Na^+^ was replaced with NMDG (Fig. 5C). Both for the wt NALCN and the two CLIFAHDD mutants, the time-dependent decay of the Na^+^-dependent component of the NALCN current displayed an inactivation-like component in the −80 mV/−20 mV range of the membrane potential that was best fitted with a single exponential time-constant (Fig. 5E). Of note, kinetics of this time-dependent decay at −40 mV was 3 and 4 times slower, respectively, for the L509S mutant (98.84 ± 10.80 ms) and for the Y578S mutant (130.65 ± 7.65 ms), compared to that measured for the wt NALCN current (30.66 ± 3.60 ms).

### No change in sodium selectivity for NALCN current potentiated in low extracellular Ca^2+^ concentration

**Figure 6:**
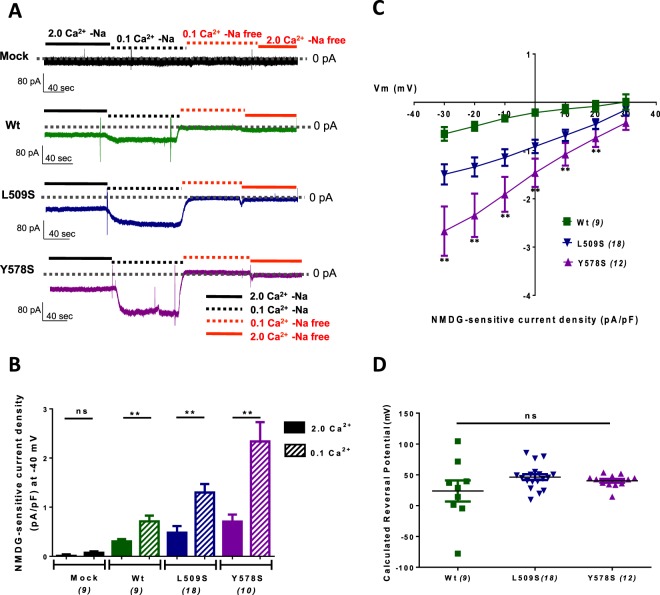
The NALCN current for wt, Y578S and L509S variants is potentiated in low extracellular Ca^2+^ with no change in reversal potential. **(A**) Representative traces obtained on cells held at −40 mV (HP) during application/wash of an extracellular Na^+^-free solution (replaced with NMDG) with high (2 mM) or low (0.1 mM) extracellular Ca^2+^ for a mock (NLF1)-transfected cell, a NALCN/NLF1 (wt)-transfected cell, a NALCN CLIFAHDD mutant (L509S)/NLF1-transfected cell, and a NALCN CLIFAHDD mutant (Y578S)/NLF1-transfected cell. (**B**) Density of the external Na^+^-dependent background current (Na^+^-free) with high (2 mM, filled bars) or low (0.1 mM, hatched bars) extracellular Ca^2+^ for mock (NLF1)-transfected cells (−0.008 ± 0.029 pA/pF in [Ca^2+^]_e_ = 2 mM and −0.073 ± 0.028 pA/pF in [Ca^2+^]_e_ = 0.1 mM, n = 9, P = 0.136), NALCN/NLF1 (wt)-transfected cells (−0.303 ± 0.046 pA/pF in [Ca^2+^]_e_ = 2 mM and −0.712 ± 0.115 pA/pF in [Ca^2+^]_e_ = 0.1 mM, n = 9, p = 0.004). NALCN CLIFAHDD mutant (L509S)/NLF1-transfected cells (−0.529 ± 0.148 pA/pF in [Ca^2+^]_e_ = 2 mM and −1.347 ± 0.188 pA/pF in [Ca^2+^]_e_ = 0.1 mM, n = 16, P = 0.001), and NALCN CLIFAHDD mutant (Y578S)/NLF1-transfected cells (−0.792 ± 0.126 pA/pF in [Ca^2+^]_e_ = 2 mM and −2.466 ± 0.415 pA/pF in [Ca^2+^]_e_ = 0.1 mM, n = 16, P = 0.001). Statistical significance was calculated with an unpaired student’s t test (**C**) I/V relationship in the −30 mV to + 30 mV range of the Na^+^-dependent component recorded in low concentration of extracellular Ca^2+^ (0.1 mM) of wt NALCN (n = 8), NALCN-Y578S (n = 12) and NALCN-L509S (n = 18) currents. (**D**) Mean reversal potential (E_Rev_) of the Na^+^-dependent component of wt NALCN (E_rev_ = 36.55 ± 12.91 mV, n = 8), NALCN-Y578S (E_rev_ = 40.22 ± 2.99 mV, n = 12) and NALCN-L509S (E_rev_ = 46.22 ± 4.70 mV, n = 18) currents. Each dot represents the E_Rev_ of 1 cell. For each cell, the E_Rev_ was calculated from the linear part of its I/V relationship as illustrated in (**C**). Statistical significance was calculated with a Tukey’s multiple comparisons test.

An interesting feature of the NALCN current is its sentivity to extracellular Ca^2+^. Indeed, a decrease of extracellular Ca^2+^ from 2 mM to 0.1 mM resulted in a strong enhancement of the NALCN current in native neurons^15,16^. Therefore, we chose to explore the sensitivity to extracellular Ca^2+^ of the wt, Y578S and L509S NALCN channels expressed in NG108-15 cells (Fig. 6). As illustrated in Fig. 6A, the reduction of extracellular Ca^2+^ resulted in a marked increase in the Na^+^-background current in cells expressing the wt, Y578S and L509S NALCN channels, but not in the control (mock) condition. In average, the NALCN current density was 2 to 3 fold higher in these three conditions when the extracellular Ca^2+^ concentration was reduced from 2 mM to 0.1 mM (Fig. 6B). We next examined the −30 to +30 mV range of the I/V relationship of the NMDG-sensitive current in cells expressing either wt, L509S or Y578S channels (Fig. 6C) in order to estimate the reversal potential of the corresponding currents. The reversal potential was calculated from the I/V curve of each cell. The mean values of the reversal potentials of the NMDG-sensitive current for wt, Y578S and L509S NALCN channels showed no significant difference (wt NALCN, E_rev_ = 36,55 ± 12,91 mV, n = 8; L509S NALCN, E_rev_ = 46.22 ± 4.70 mV, n = 18; Y578S NALCN, E_rev_ = 40.22 ± 2.99 mV, n = 12; Fig. 6D). These findings indicate that the selectivity to Na^+^ of NALCN channel was not significantly altered by the L509S and Y78S mutations.

### A difference in membrane expression does not appear to account for the gain-of-function effect of L509S and Y578S pathogenic variants

**Figure 7:**
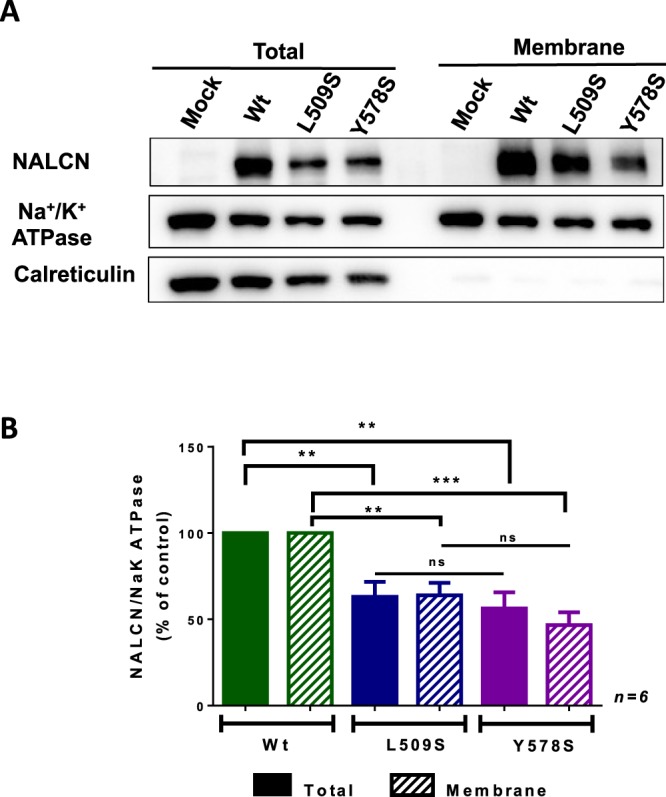
Total and membrane expression of wt NALCN channel and its L509S and Y578S variants. (**A**) Representative cell surface biotinylation experiment. The cell surface biotinylation assay was performed in cells transfected with NLF1 alone (Mock) or along with wt NALCN, NALCN-L509S or NALCN-Y578S (GFP tagged). The Na^+^/K^+^ ATPase, a plasma membrane protein, and the calreticulin, a reticulum-resident protein, were used internal controls. (**B**) The expression levels of wt NALCN, NALCN-L509S and NALCN-Y578S were normalized with the Na^+^/K^+^ ATPase level from both total and membrane fractions. The total expression compared to NALCN was: L509S, 62.99** ± **8.725%, p < 0.01; Y578S, 56.24 ± 9.408%, p < 0.01, while the membrane expression compared to NALCN was: L509S, 63.94** ± **7.261%, p < 0.01; Y578S, 46.69 ± 7.412%, p < 0.001 (n = 6 independent experiments). Statistical significance was calculated with a one-way ANOVA multiple comparisons test.

We next performed the cell biotinylation of surface proteins in order to determine whether the gain-of-function effect of the L509S and Y578S NALCN mutants could result from an increase in plasma membrane expression (Fig. 7). Six independent experiments were conducted and a representative one is presented in Fig. 7A. We found that both the total expression and the membrane expression of the L509S and Y578S NALCN mutants (normalized with the Na^+^/K^+^ ATPase expression level) were significantly lower than expression of wt NALCN. In average (n = 6), the total expression normalized to wt NALCN was 62.99** ± **8.72% for L509S (p < 0.01) and 56.24 ± 9.41% for Y578S (p < 0.01). The membrane expression normalized to wt NALCN was 63.94 ± 7.26%, for L509S (p < 0.01) and 46.69 ± 7.41% for Y578S (p < 0.001). These data suggest that the observed gain-of-function effect of L509S and Y578S NALCN mutants does not result from an increased membrane expression but rather a functional enhanced activity as identified in patch-clamp experiments.

## Discussion

This study describes the electrophysiological properties of recombinant NALCN channel expressed in the neuronal cell line NG108-15. In good agreement to that previously reported, the inward NALCN current was carried out by Na^+^ ions, blocked by Gd^3+^, insensitive to TTX and potentiated by lowering external Ca^2+^ concentration. Notably, NALCN expression in NG108-15 cells revealed novel functional properties. First, the NALCN current measured during hyperpolarizing pulses displayed inactivation-like process and a non-linear current-voltage relationship. Second, exploring representative CLIFAHDD and IHPRF1 mutations revealed that these NALCN variants showed gain- and loss of NALCN channel activity, respectively.

NALCN is mainly expressed in neurons, especially of the central nervous system (http://www.mousebrain.org/)^52^. It was therefore important to set-up the study of the functional properties of the wt and pathogenic variants of NALCN channel in a neuron-like environment. Functional expression of recombinant NALCN was first described using HEK293 cells^3,12,32,50,51^, but the relevance of this cell model to express NALCN has been disputed^53,54^. The pionier studies by Lu and colleagues, using HEK293 cells, have established the main NALCN electrophysiological signature, i.e. the ‘sodium leak’ properties^3,12,15^. The few other cellular models used to express NALCN, such as the pancreatic β MIN6 cell line^13^ and the undifferentiated SH-SY5Y neuronal cell line^15^ have confirmed some electrophysiological properties and revealed additional ones. Indeed, NALCN does not exhibit leak activity in MIN6 cells but rather an inactivating Na^+^ current activated by a G-protein coupled receptor (GPCR) that was evidenced following acetylcholine activation of muscarinic receptors^13^. In NG108-15 cells, we demonstrate that combined expression of the recombinant NALCN and NLF1 proteins was sufficient to record a Na^+^ background current. Importantly, the Unc79 and the Unc80 ancillary subunits are endogenously expressed in NG108-15 cells, indicating that this neuronal cell line, in which we recorded a Na^+^ background current, expressed the necessary components of the NALCN channelosome. Of note, the NALCN-related background Na^+^ current was recorded only in NALCN- and NLF1-transfected NG108-15 cells differentiated in neuron-like cells, suggesting that neuronal differentiation favors the functional expression of NALCN, possibly also by stimulating the functional expression of additional components of the neuronal NALCN channelosome.

An important finding of this study is that the Na^+^ background current related to heterologous NALCN expression in differentiated NG108-15 cells exhibits electrophysiological properties that have not been described elsewhere. The inward NALCN current triggered by hyperpolarizing pulse in the negative range of membrane potential (<−20 mV) displayed a time-dependent decay of the current amplitude, reminiscent of the voltage-dependent inactivation process of Na^+^ and Ca^2+^ voltage-gated channels. This inactivating current was not observed in Mock-transfected cells and was eliminated in NALCN-transfected cells by the removal of external Na^+^, indicating that it was linked to NALCN expression. To the best of our knowledge, such an inactivation process was never observed in the previous NALCN current recordings, especially those obtained from the HEK293 cell line that represent the vast majority of the electrophysiological data on recombinant NALCN^3,12,32,50,51^. This time-dependent decay of the background current is clearly attributable to the functional expression of NALCN as it was observed for both the wt and the CLIFAHDD mutants. Importantly, a significantly distinct time-course was found for the CLIFAHDD currents, which were 3 to 4 fold slower than the wt NALCN current. Also, the current-voltage (I/V) relationship of the NALCN current recorded in differentiated NG108-15 cells was not linear, contrary to that reported in published studies describing NALCN expression in HEK293 cells. These findings reveal novel electrophysiological properties of NALCN when functionally expressed in differentiated neuronal cells. It remains however important to determine if the electrophysiological properties of NALCN channel in differentiated NG108-15 cells are relevant for native neurons.

Another major finding of this study was the functional characterization of several pathogenic NALCN variants. Expression of NALCN carrying the W1287L missense mutation found in 3 IHPRF1 siblings^17^ did not result in any detectable Na^+^ background current. IHPRF1 is recessively inherited and patients carried either frameshift or missense mutations, suggesting loss-of-function mutations of NALCN. Our findings describing a loss of channel activity for the W1287L mutation of NALCN are therefore in good agreement with the genetics data. On the contrary, functional expression of the two missense mutations found in CLIFAHDD patients, L509S and Y578S, revealed a gain-of-function effect. Compared to wt NALCN, expression of the two CLIFAHDD variants showed Na^+^ background current of significantly higher current density. In addition, a significant slowing of the current inactivation was observed, potentially contributing also to the gain-of-function effect. The gain-of-function effect of L509S and Y578S mutations likely results from a functional alteration instead of a trafficking change since no increase, but rather a decrease, in plasma membrane expression was detected by a cell surface biotinylation assay for the two CLIFAHDD variants. Although we found no evidence for a change in Na^+^ selectivity or in the 2-3 fold potentiation in low external Ca^2+^ for the two mutants, other electrophysiological or modulation properties yet to be identified could also contribute to the gain-of-function effect of L509S and Y578S mutations.

Our data are in agreement with previous studies that suggested that pathogenic variants of NALCN linked to CLIFAHDD are gain-of-function mutations. As a matter of fact, most of the mutations found in CLIFAHDD patients localize in the transmembrane segments S5 and S6 of domains I, II, III and IV. These segments are well known to form the pore of the four-domain ion channels. Semi-dominant missense mutations found in the S6 of domain II of NCA-1 (A596V/A717V and D600E), the *C*. *elegans* ortholog of mammalian NALCN, result in an uncoordinated, and exaggerated body bends phenotype during spontaneous or stimulated locomotion (e.g. « coiler » phenotype)^55^. This contrasts with loss-of-function NCA-1 mutations where animals are fainters that fail to sustain sinusoidal locomotion and succumb to long periods of halting^55–57^. This led to the conclusion that A596V and D600E are gain-of-function mutations. In addition, synaptic calcium transients are significantly reduced in *Nca* loss-of-function mutants and increased in *Nca* gain-of-function mutants^55^. Another mutation was also described in the *Nalcn* gene in the *dreamless* mutant mouse^50^. This mutation is dominant and results in the N315L substitution in helix S6 of domain I, which is conserved among vertebrates and invertebrates. Functional expression of the N315L mutant of NALCN in HEK293 cells was found to result in a leak current significantly higher than for the wt NALCN^50^. Another argument that favors the gain-of-function hypothesis for the CLIFAHDD mutations comes from the fact that the EMG in a L590F patient revealed abnormalities that indicate a possible motor neuron/axon hyperexcitability^27^. Of note, the pan-neuronal expression of the R1230Q mutation in the NCA-1 of *C*. *elegans* that reproduces the R1181Q found in 3 patients with CLIFAHDD induced a coiling locomotion identical to that of the gain-of-function *nca-1* A596V/A717V mutant^22^. Altogether, our data validate the hypothesis that at least some mutations found in patients with the CLIFAHDD syndrome are gain-of-function mutations.

Most of the recessive IHPRF1 (NALCN) and IHPRF2 (Unc80) mutations are predicted to result in non-functional proteins. Since we could not identify NALCN current with a NALCN variant carrying an IHPRF1 W1287L mutation in our experimental conditions, our conclusion is that this IHPRF1 missense variant of NALCN also results in a non-functional channel. It is expected that the lack of NALCN activity should lead to cellular/neuronal hyperpolarization^48^ and consequently a decrease in the firing properties of these cells, as observed when *Nalcn* is knocked out or knocked down^3–10^. Conversely, the two mutations found in CLIFAHDD patients, L509S and Y578S, clearly induced a gain of channel activity in the neuronal NG108-15 cells. It is predicted that higher activity of a Na^+^ background current should result in a more depolarized RMP, possibly in an increase of the firing properties of the NALCN-expressing cells as described in neurons from the deep mesencephalic nucleus of the *dreamless* mutant mouse^50^. However, a significant increase in NALCN activity could worsen cellular excitability by switching the RMP to depolarized state, attenuating firing activity. Strikingly, a study in which the Y578S (Y621S in *C*. *elegans*) dominant mutation was reproduced in the NCA-1 channel of *C*. *elegans* revealed a locomotor phenotype reminiscent to a loss-of-function mutation (e.g. fainting behavior). The same study reported a gain-of-function phenotype for the L509S (L556S in *C*. *elegans*) dominant mutation, e.g. coiling behavior and hypersensitivity to aldicarb^27^. The authors concluded that three genetic mechanisms could give rise to NALCN channelopathies: (1) NALCN loss-of function in IHPRF, (2) gain-of-function and (3) dominant-negative in dominantly inherited CLIFAHDD^27^. This conclusion was based on the locomotor behavior and the albicarb sensitivity of *C*. *elegans* models. Our results, at the channel/cellular level, support the two first hypothesis. First, we describe that the IHPRF mutation induces loss of NALCN channel activity. Second, we describe that both the Y578S and the L509S CLIFAHDD variants are gain-of-function mutants. In addition, we report that the Y578S variant exhibits a more pronounced gain of channel activity than the L509S variant. It is tempting to speculate that the Y578S variant would depolarize the cells to a greater extend than the L509S variant. Such a large depolarization with the Y578S mutant would result in a decrease in excitability in cell types highly depending on NALCN activity, mimicking the IHPRF loss-of-function phenotype. Such a molecular mechanism might explain why IHPRF patients and CLIFAHDD patients share several, but not all, symptoms.

To the best of our knowledge, it is the first report of functional effects of IHPRF and CLIFAHDD variants of NALCN. Although the precise mechanisms involved in the electrophysiological defects remain to be clarified, the findings reported here validate the neuronal cell line NG108-15 to investigate the functional properties of NALCN variants. Using a *C*. *elegans* model mimicking IHPRF, it was recently suggested that NALCN deficiency may be corrected by pharmacological targeting of other channels^58^. Conversely, one may predict that partial inhibition of NALCN could present some benefits for CLIFAHDD patients. Cellular systems expressing IHPRF and CLIFAHDD variants are therefore of interest for further pharmacological investigations. As a conclusion, our present work provides the first report of functional impact of pathogenic variants of NALCN found in the two-associated and devastating human diseases, and paves the way to the identification of therapeutical strategies to treat NALCN-related diseases.
